# Saikosaponin D Rescues Deficits in Sexual Behavior and Ameliorates Neurological Dysfunction in Mice Exposed to Chronic Mild Stress

**DOI:** 10.3389/fphar.2021.625074

**Published:** 2021-02-16

**Authors:** Zhuo Wang, Jianwei Li, Wei Wu, Tao Qi, Zhansen Huang, Bo Wang, Shixiong Li, Chen Li, Jiuyang Ding, Yuanning Zeng, Peng Huang, Zhihua Zhou, Yanjun Huang, Jian Huang, Xiaohan Wang, Qiyuan Huang, Guanghuan Zhang, Pingming Qiu, Jun Chen

**Affiliations:** ^1^ Department of Infertility and Sexual Medicine, The Third Affiliated Hospital of Sun Yat-sen University, Guangzhou, China; ^2^ School of Forensic Medicine, Southern Medical University, Guangzhou, China; ^3^ School of Forensic Medicine, Guizhou Medical University, Guiyang, China; ^4^ Research Center for Good Practice in TCM Proessing Technology, Guangdong Pharmaceutical University, Guangzhou, China; ^5^ Foshan Maternal and Child Health Hospital, Affiliated Hospital of Southern Medical University, Foshan, China; ^6^ Department of Neurology, The First Affiliated Hospital, School of Clinical Medicine of Guangdong Pharmaceutical University, Guangzhou, China; ^7^ Department of Neurology, Zhujiang Hospital, Southern Medical University, Guangzhou, China; ^8^ School of Laboratory Medicine and Biotechnology, Southern Medical University, Guangzhou, China; ^9^ Department of Nutrition, Hospital of Integrated Traditional Chinese Medical and Western Medicine, Southern Medical University, Guangzhou, China

**Keywords:** saikosaponin d, medial posterodorsal amygdala, sexual behaviour, emotion, stress

## Abstract

Often associated with sexual dysfunction (SD), chronic stress is the main contributing risk factor for the pathogenesis of depression. Radix bupleuri had been widely used in traditional Chinese medicine formulation for the regulation of emotion and sexual activity. As the main active component of Radix bupleuri, saikosaponin D (SSD) has a demonstrated antidepressant effect in preclinical studies. Herein, we sought to investigate the effect of SSD to restore sexual functions in chronically stressed mice and elucidate the potential brain mechanisms that might underly these effects. SSD was gavage administered for three weeks during the induction of chronic mild stress (CMS), and its effects on emotional and sexual behaviors in CMS mice were observed. The medial posterodorsal amygdala (MePD) was speculated to be involved in the manifestation of sexual dysfunctions in CMS mice. Our results revealed that SSD not only alleviated CMS-induced depressive-like behaviors but also rescued CMS-induced low sexual motivation and poor sexual performance. CMS destroyed astrocytes and activated microglia in the MePD. SSD treatment reversed the changes in glial pathology and inhibited neuroinflammatory and oxidative stress in the MePD of CMS mice. The neuronal morphological and functional deficits in the MePD were also alleviated by SSD administration. Our results provide insights into the central mechanisms involving the brain associated with sexual dysfunction. These findings deepen our understanding of SSD in light of the psychopharmacology of stress and sexual disorders, providing a theoretical basis for its potential clinical application.

## Introduction

Stressful stimuli are aplenty in modern society, and stressful life events are the main cause of depression (Hammen, 2018). Stress-related mood disorders have become a serious public health problem and drawn much attention (Becker and Kleinman, 2013; Ménard et al., 2016). Another great concern frequently overlooked is that depressed male patients who experience chronic stress often show reduced sexual interest, have difficulty with sexual arousal, and exhibit poor sexual function (Hosain et al., 2013; Breyer et al., 2014; Deumic et al., 2016). Indeed, the prevalence of these problems are much higher in depressed men than in the general population (El Yazidi et al., 2019). In experimental studies, chronic stress has also been reported to induce reduced sexual motivation, testicular injury, and nonorganic erectile dysfunction in male animals (Hou et al., 2014; Chen et al., 2019). Depression mainly occurs in young and middle-aged people (Bogren et al., 2018); sexual dysfunction in these men has a huge negative impact on the individuals’ and couples’ quality of life.

As an important component of the limbic system (Sokolowski and Corbin, 2012), the amygdala plays a key role in responding to emotional stimuli; its involvement in the pathophysiological response to stress had been well recognized (Drevets, 2003; Barry et al., 2017; Kedo et al., 2018). In addition, the amygdala also plays an important role in the arousal and execution of sexual behavior (Kühn and Gallinat, 2011; Seok et al., 2016). Direct bilateral electrical damage to the medial amygdala causes abnormal emotional behaviors in animals (Vinkers et al., 2010). The mating behaviors of male rats with a medial amygdala injury were severely impaired, while that of the rats with a basolateral amygdala injury remained unchanged (Kondo, 1992). The medial amygdala plays a dual role in both emotional processing and regulation of sexual behavior. The posterodorsal medial amygdala (MePD) is a subnucleus of the medial amygdala that expresses sex hormone receptors and is involved in regulating moods and sexual behavior (Rasia-Filho et al., 2012; Bergan et al., 2014). It can exert a regulatory effect on sexual motivation through its connection with downstream nuclear clusters (Holder and Mong, 2017). Sexual dysfunction in male patients with chronic stress-induced depression may be caused by a dysfunctional MePD in the central nervous system (CNS).

Common antidepressants have a long onset of action, accompanied by side effects of varying severity (Tollefson, 1991). Sexual dysfunction is one of the most commonly reported adverse effects of antidepressants (Montejo et al., 2019). Only few alternatives or supplemental drugs have been found to effectively alleviate the abovementioned symptoms. Therefore, there is an urgent need to identify and develop drugs that can clinically improve emotional dysfunctions, without disrupting sexual activity. Radix bupleuri is a widely used ingredient in traditional Chinese medicine (TCM) formulations for relieving depression. It plays a therapeutic role in the Xiaoyaosan (Liu et al., 2015), Sinisan (Wang et al., 2020), Chaihu-Shugan-San (Qiu et al., 2014), and other traditional antidepressant Chinese medicine formulae. Modern pharmacological studies have shown that saikosaponins are the main bioactive component of Radix bupleuri accounting for its antidepressant effects (Chao et al., 2020). Saikosaponin D (SSD) had a demonstrated antidepressant effect in multiple depression models (Li et al., 2017; Xu et al., 2019; Chao et al., 2020). However, it remains unknown whether SSD could ameliorate chronic stress-induced impairments of sexual activity. This study sought to explore the effect of SSD on chronic stress-induced depression and symptoms related to sexual dysfunction in male mice and elucidate its potential central mechanisms.

## Material and Methods

### Animals

C57 BL/6J adult male mice, aged 8–10 weeks, were provided by the Laboratory Animal Center of Southern Medical University (Guangzhou, Guangdong). All animals were raised in a single cage in a standard specific pathogen-free experimental environment (12 h light/dark cycle, with *ad libitum* access to dry food and clean water). The indoor temperature was maintained at 23 ± 1°C, and the humidity was maintained at 50–60%. Animal treatments, including anesthesia induction and euthanasia, were conducted in accordance with the Principle of Laboratory Animal Care (NIH Publication no. 85–23, revised 1985). All experimental procedures were conducted in accordance with the requirements of the China Animal Ethics Committee and were approved by the Animal Ethics Committee of The Third Affiliated Hospital of Sun Yat-sen University.

### Chronic Mild Stress (CMS) Modeling and Drug Administration

In addition to those included in the control group, the remaining mice were subjected to chronic unpredictable stress. The procedures for CMS model development were adapted from a previous study (Qin et al., 2019). The stress paradigm included fasting, day/night reversal, forced swimming, noise, stroboscopy, restraint stress, water deprivation, and a wet cage; these stressors were randomly subjected 2–3 times every day. The unpredictable stimuli were administered in different ways for 7 weeks. After chronic stress stimulation for 3 weeks, the CMS mice were then randomly assigned to the SSD treatment group (CMS+SSD) or nontreated CMS group (CMS). The SSD dosage was adopted from previous validated research (Xu et al., 2019; Chao et al., 2020) (M3936, Abmole, Houston, TX, United States, >98%, 1 mg/kg; dissolved in saline and administered via gavage) and the treatment lasted three weeks; in contrast, the mice in the CMS group were treated with saline. In the preliminary experiment, we had used 1 mg/kg dosage in age-matched mice to verify the potential adverse effects of SSD. The SSD treatment had little effect on depressive and sexual behaviors of the mice, after which we performed the following experiments. All experiments were strictly implemented in accordance with the *3R* principles; each group had 20 mice. At the end of the CMS modeling and SSD treatment, the animals were used for behavioral, morphological, electrophysiological, and biochemical analyses. All animals were assigned a numerical code and the investigators were always blinded to the treatment groups until the completion of data analysis.

### Sucrose Preference Test

The sucrose preference test is the main method of testing the core symptom of CMS depression model-anhedonia (Cao et al., 2013). After 23 h of fasting and water abstinence, each mouse was provided with two bottles of water (weights measured in advance): one bottle contained 1% sucrose water and the other had pure water. Pure water consumption and sugar water consumption of the mice were calculated after 1 h. The percentage of sugar water consumption was calculated in terms of the total weights of liquid consumed.

### Forced Swimming Test (FST)

The mice were placed in a swimming bucket filled with water at 24 ± 2°C, with a depth of 20 cm, for 6 min. The time the mice spent in immobility within the last 4 min was recorded. The duration of immobility was recorded when mice ceased struggling.

### Y-Maze Preference Test

This experiment was conducted in a closed Y-shaped maze as previously described (Petrulis and Johnston, 1999). The maze was made of PVC plastic and consisted of three long arms and perforated plexiglass boxes were placed at the ends of two top arms. The animals were allowed to explore the animals behind the transparent perforated plexiglass boxes. The mice were allowed to move freely in the maze for 5 min, and the time spent exploring each end of the Y-maze was recorded.

### Sexual Motivation Test

In reference to research on the sexual motivation of rats (Chu and Ågmo, 2016), we established a mouse model for the sexual motivation test. The experimental device used is similar to that used in rats. The equipment consisted of a rectangular open field having two chambers with openings on the diagonally opposite walls. The front of the chamber is made of a wire mesh, which allows the animals to see, smell and touch the animals in the chamber. Male and female animals are respectively placed in the two chambers. The virtual area in front of the cage is the motivation exploration area. A camera was suspended to track the movements of the animals, and the preference score was defined by female motivation area time/(female motivation area time + male motivation area time).

### Sexual Behavior Test

The sexual interaction test was adapted from a previous study (Benelli et al., 2002). Briefly, the experiment was conducted in a quiet, dimly lit environment at nightphase. After three days of preadaptation to the experimental arena (20 min per day; arena area, 40 cm*40 cm*40 cm), the tested male mice were allowed to interact with aphrodisiac female mice (Grønli et al., 2005) for 30 min. The recorded masculine sexual behavior include the latency to first mount, mount frequency, intromission frequency, and the ratio of intromission and total mount.

### Immunofluorescence Staining

Following paraformaldehyde perfusion and fixation, the mouse brains were dehydrated with 30% sucrose. After cryofixation and obtaining cryosections of the whole brain, the brain slices were incubated in 5% bovine serum albumin containing 0.5% Triton X-100 for 1 h, after which they were treated with the following primary antibodies: rabbit polyclonal anti-Iba1 (1:300; ab153696; Abcam) and rabbit polyclonal anti-glial fibrillary acid protein (GFAP) (1:300; ab7260; Abcam); the primary antibodies were incubated at overnight at 4°C. On the next day, following three washes with 0.1M phosphate-buffered saline (PBS), the slices were incubated with secondary antibody at room temperature for 1.5 h, mounted with DAPI-containing mounting medium, and sealed after washing again with PBS. Fluorescent images were acquired by confocal microscopy (Leica, Wetzlar, Germany) and analyzed using the Image J software.

### Western Blotting

The brain protein samples were extracted using RIPA buffer containing a protease inhibitor cocktail (Roche, Basel, Switzerland), and the extracted proteins were electrophoresed in a 10–12% sodium dodecyl sulfate-polyacrylamide gel (Willget, China). Thereafter, the separated proteins were transferred onto polyvinylidene difluoride membranes; the membranes were then probed overnight with the following primary antibodies: mouse polyclonal anti-GFAP (1:800; cat# 3670; Cell Signaling Technology, MA, United States), rabbit polyclonal anti-MAP2 (1:800; cat# 4542; Cell Signaling Technology) and mouse polyclonal anti-GAPDH (1:2,000, sc-365062, Santa Cruz Biotechnology, TX, USA). On the next day, after washing, the membranes were incubated with horseradish peroxidase (HRP)-conjugated secondary antibody for 1 h at room temperature. The proteins were then visualized using an enhanced chemiluminescent substrate (ECL; Thermo Fisher Scientific, IL, United States).

## Enzyme-linked immunosorbent assay (ELISA) Determination of IL-1β and IL-6

After behavior tests, the mice were sacrificed by cervical dislocation and MePD brains tissue were rapidly isolated on ice. The isolated brain tissue were homogenized in ice-cold phosphate buffer (pH 7.4) and centrifuged at 10, 000 g at 4°C for 15 min. The supernatants were collected and 100 µl supernatants were added in the plates to measure IL-1β and IL-6 with commercial ELISA kits (DY401 and DY406 respectively; R&D Systems, Minneapolis, United States) in accordance with the manufacturer’s instructions. The optical density (OD) was measured with a microplate reader (PerkinElmer, Waltham, MA, United States) at a wavelength 450 nm and normalized to total soluble protein concentration with a BCA Protein Assay Kit (Cell Signaling Technology).

### Reactive Oxygen Species (ROS) Measurement

Oxidative stress in the MePD was evaluated by determining the generation of ROS. Quantitative measurements of ROS were performed using a double-sandwich ELISA method (ROS assay kit; QYE2656; Qualityard, Beijing, China) in accordance with the manufacturer’s instructions. The OD value of each hole was measured sequentially with a microplate reader (PerkinElmer) with the wavelength of 450 nm and the values were expressed relative to the signal of controls.

### Stereotaxic Surgery

Stereotaxic surgeries were conducted as previously described (Wang et al., 2019b). The mice were anesthetized with intraperitoneal injection of 4% pentobarbital sodium (40 ml/kg) and placed on a stereotaxic apparatus (RWD, Shenzhen, China). The scalp was shaved, and the skin was disinfected with 75% alcohol. The skin was cut along the median line of the exposed bone, and the subcutaneous tissue was separated to fully expose the skull. After drilling through the skull, above the MePD (AP -1.7; ML 2.0; DV -5.0) nucleus, adeno-associated viruses (AAV) expressing the green fluorescent protein (GFP; BrainVTA, Wuhan, China) was injected into the target site.

### Confocal Image Analysis of Spine Density

The GFP-stained brain sections were observed under a confocal microscope (LSM510, Meta, Zeiss). For dendritic spine density analysis, z-series stacks were obtained with 10 consisted scans at a high zoom at 1 mm intervals in the Z-axis. The dendritic spines were sampled from 3–5 distal dendrites of all GFP-positive neurons. The density of dendritic spines in the MePD was measured using Image J software.

### *In vivo* Electrophysiological Recordings

After the mice were placed on a stereotaxic apparatus and the skull was exposed, a glass electrode was slowly lowered toward the MePD to record neuronal activity. An electrical stimulation was delivered (STG4008 stimulator, Multichannel Systems, Germany) through the electrode—intensity, 0.1–0.9 mA; duration, 0.2 ms; frequency, 0.2–40 Hz. At the end of the experiments, only the data collected from the right stimulation sites were used for analyses.

### Statistics

The GraphPad Prism 7.0 software was used to analyze the data and create the figures. Data are presented as mean ± standard error of mean (SEM) and analyzed using a Student’s *t*-test or an analysis of variance, according to the different experiments. The significance level for all tests was set to p/q < 0.05.

## Results

### SSD Alleviated CMS-Induced Depressive-like Behaviors in Mice

**FIGURE 1:**
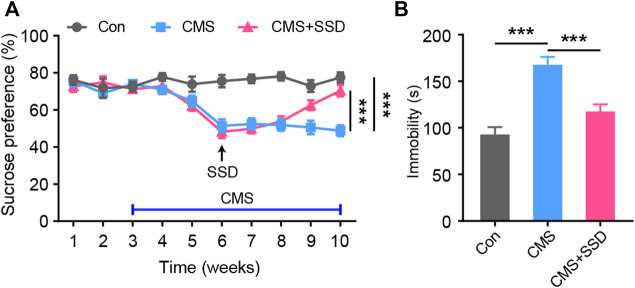
The administration of saikosaponin D (SSD) induced antidepressant-like effects in chronic mild stress (CMS) mice. **(A)** Schematic of the experimental design; we used sucrose preference test to assess the depression-like behaviors of mice on a weekly basis. (*n* = 10, repeated-measures analysis of variance [ANOVA]). **(B)** Effects of SSD on depression-type behavior determined via forced swimming test (*n* = 9–11, one-way ANOVA). Data are presented as the mean ± standard error of mean. ***p < 0.001.

Adult male mice underwent a seven-week CMS paradigm andthe SSD administration started from the end of the third week of CMS induction. In the sucrose preference test to evaluate anhedonia, the percentage of sucrose consumption was significantly reduced in the CMS group than in the non-stressed control group; however, SSD treatment for three weeks significantly improved sucrose consumption (Figure 1A). The results of FST revealed that the CMS group showed significantly extended duration of immobility than the non-stressed control group. In contrast, SSD administration remarkably rescued this immobility in the FST (Figure 1B). Therefore, these findings indicate that SSD demonstrated antidepressant effects in a mouse model of CMS.

### SSD Rescued CMS-Induced Low Sexual Motivation in Male Mice

**FIGURE 2:**
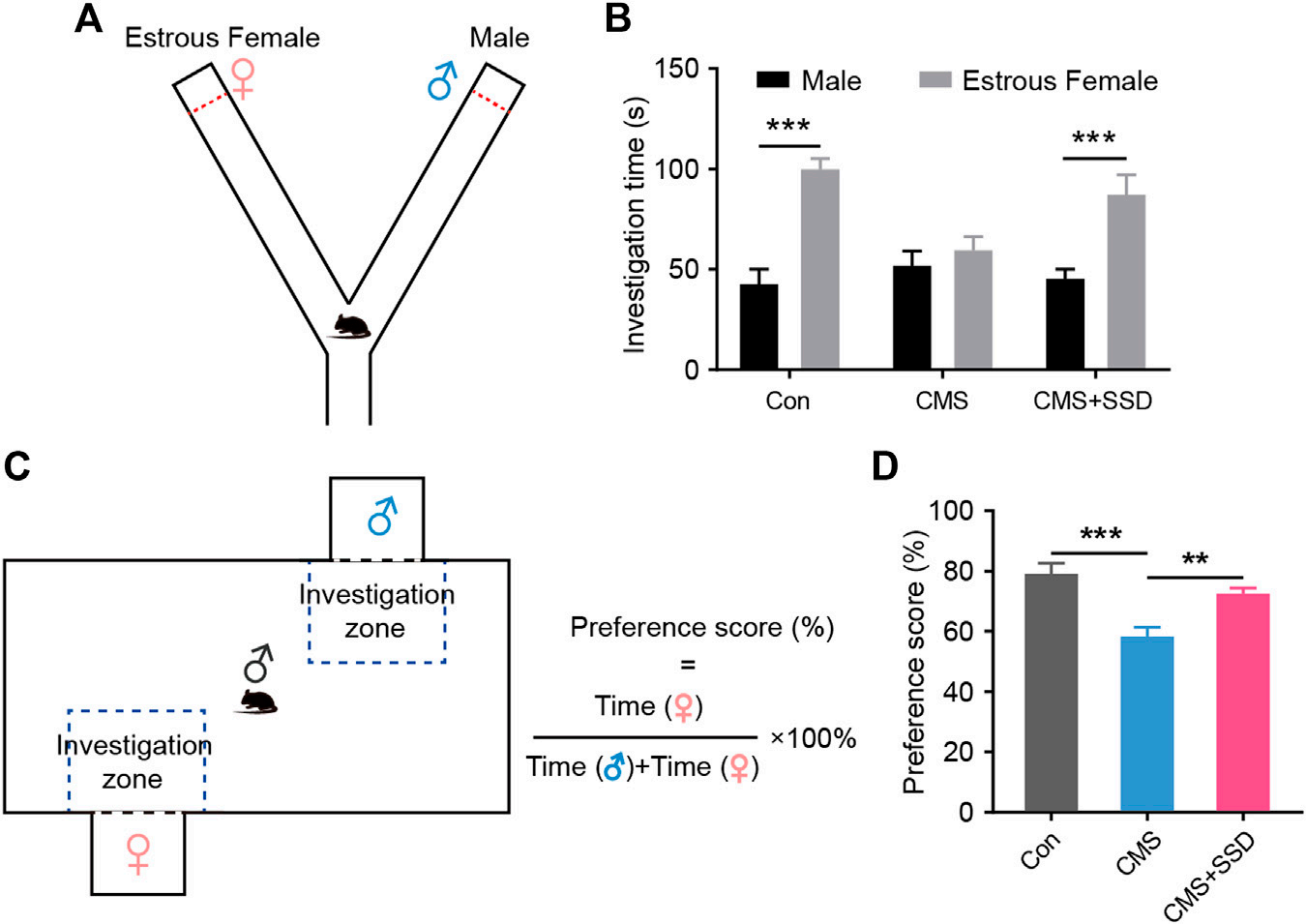
The effect of saikosaponin D (SSD) on sexual preference and sexual motivation in chronic mild stress (CMS) mice. **(A)** Schematic of the Y-maze preference test. **(B)** The time spent exploring between male and estrous female. In the experimental design, we used sucrose preference test to assess depression-like behaviors of mice on a weekly basis. (*n* = 9, Bonferroni post hoc *t*-test, analysis of variance [ANOVA]). **(C)** Schematic of the mice sexual motivation test. **(D)** Preference scores for sexual motivation. (*n* = 9, Bonferroni post hoc *t*-test, ANOVA). Data are presented as the mean ± standard error of mean. **p < 0.01, ***p < 0.001.

In a previous study, the CMS-related rodent model had shown abnormally low sexual desire (Shen et al., 2020). In a mice Y-maze sexual preference test (Figure 2A), healthy male mice preferred to explore an estrous female mouse rather than a sexually active male mouse (Figure 2B); however, the CMS mice spent nearly the same amount of time around the male/female target arm. SSD treatment remarkably alleviated the adverse effects of CMS, with a statistically significant increase in the time spent exploring an estrous female mouse than a sexually active male mouse. In another sexual motivational test adapted from a rat study (Figure 2C), when the mice were allowed to freely explore the arena, similar to our abovementioned results, the CMS-induced male mice spent less time in the female zone than those in the control group. In addition, the SSD treatment restored female exploration behavior (Figure 2D). SSD administration improved the sexual motivation of the male CMS mice.

### Effects of SSD on Sexual Performance in Male CMS Mice

**FIGURE 3:**
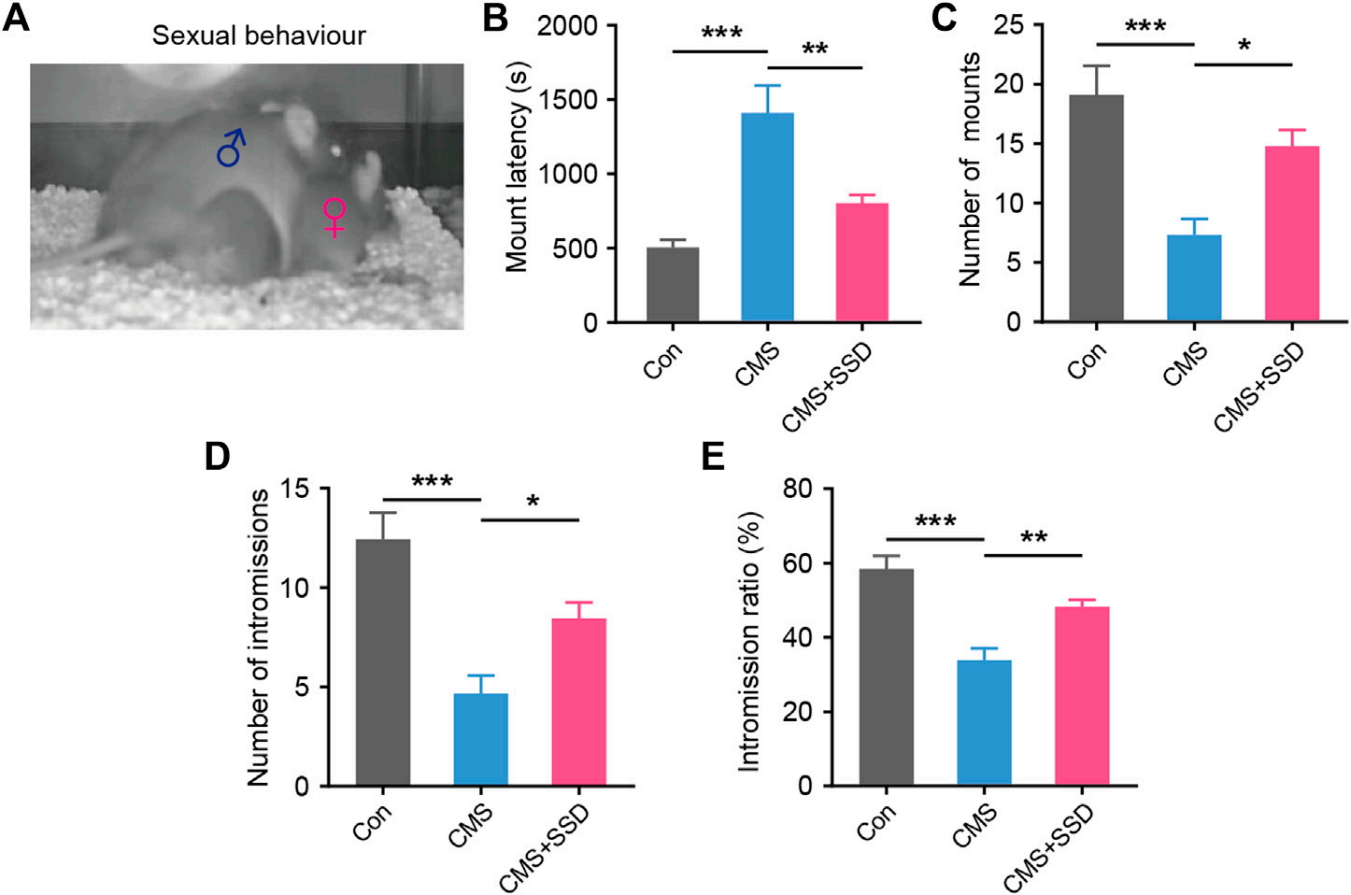
The effect of saikosaponin D (SSD) on sexual activity parameters in CMS mice. **(A)** Schematic of the sexual activity experimental. Levels of sexual behavior after three weeks’ SSD administration: **(B)** mounting latency; **(C)** total mount; **(D)** intromission frequency; **(E)** the time ratio of intromission and mount. (n = 9, one-way analysis of variance [ANOVA]). Data are presented as the mean ± standard error of mean. *p < 0.05, **p < 0.01, ***p < 0.001.

After the sexual motivation test, we performed the sexual performance test assay to further assess the sexual performance of male CMS mice. In the sexual interaction test (Figure 3A), the latency to mount, number of mounts, number of intromissions, and the ratio between intromission and mount were recorded. SSD-treated CMS mice showed significantly reduced latency for first mouth with female mouse than vehicle-treated mice (Figure 3B). This further validated our previous results of the sexual motivation test. Moreover, SSD treatment significantly increased the number of mounts and intromission (Figures 3C,D), as well as increased the intromission ratio of male CMS mice to mount a female mouse (Figure 3E). SSD administration improved the overall sexual performance of male CMS mice.

### Effects of SSD on Glial Pathology in the MePD

**FIGURE 4:**
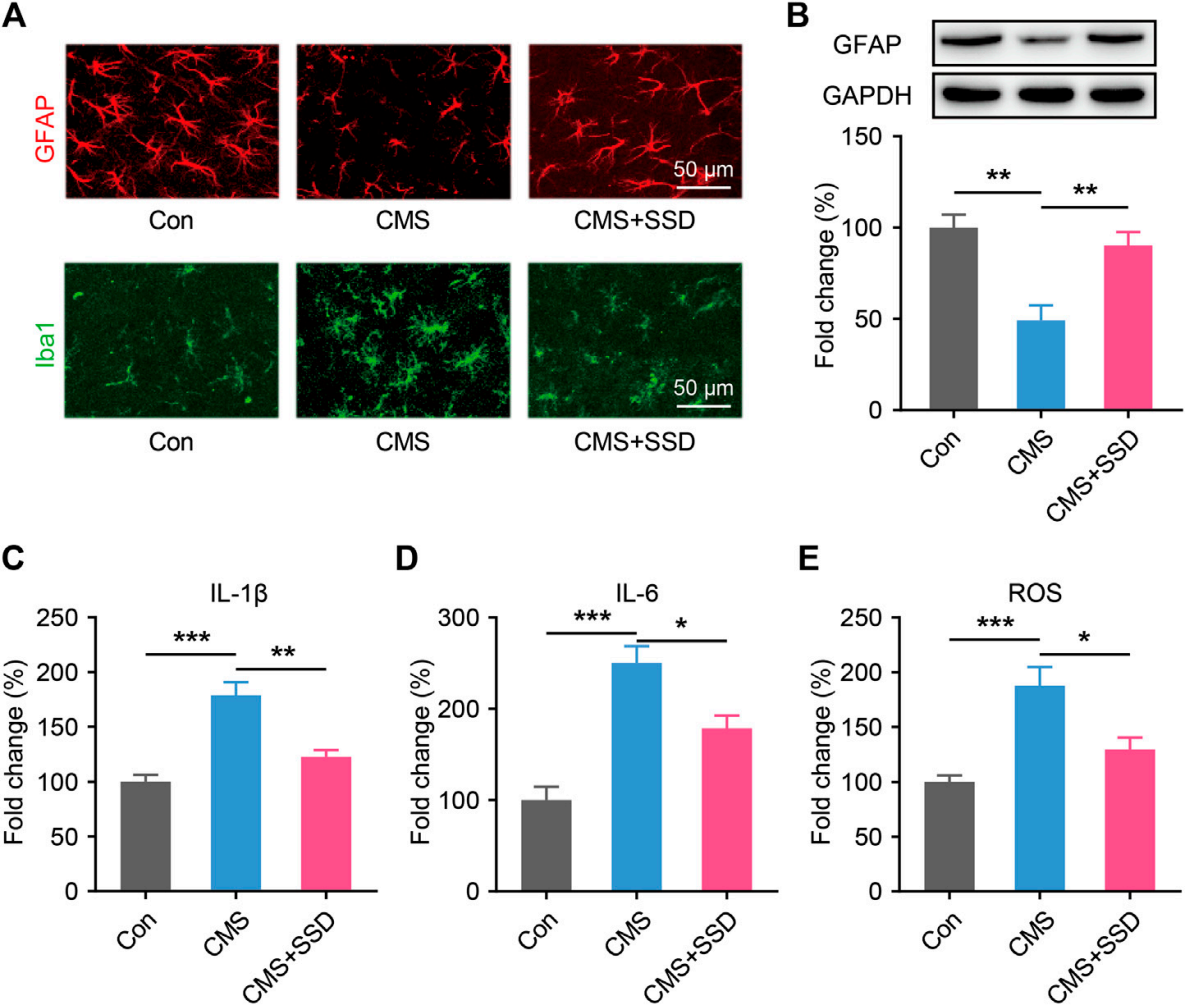
The changes in glia markers, neuroinflammation and oxidative stress in the MePD. **(A)** Astrocytes and microglia were immunofluorescent staining with glial fibrillary acid protein (GFAP; red) and Iba1 (green) after CMS and saikosaponin D treatment. (Scale bar = 50 μm). **(B)** Expression patterns of GFAP by western blotting. (*n* = 5, one-way analysis of variance [ANOVA]). **(C–E)** IL-1β, IL-6 and reactive oxygen species in the MePD were detected by enzyme-linked immunosorbent assay and presented as fold change (%). (*n* = 5, one-way ANOVA). Data are presented as the mean ± standard error of mean. *p < 0.05, **p < 0.01, ***p < 0.001.

Similar to other amygdala subareas, the MePD mainly consists of two types of cells: neurons and glial cells. As a main glial cell type in the MePD, astrocytes are sensitive to environmental stress and may be affected in terms of their morphology and functions (Tynan et al., 2013), which can impact normal neural functions. In the MePD, we observed that the GFAP + astrocytic soma and primary processes responded sensitively to stress stimuli (Figure 4A). The animals in the CMS group had a lower GFAP + astrocyte density and shrunken astrocytic morphologies than those in the control group. The results of WB further confirmed the reduced GFAP expression in the MePD of CMS mice (Figure 4B); however, these deficits were reversed by SSD administration. Furthermore, chronic stress increased the number of Iba1-positive microglia and promote their activation in the MePD of CMS mice (Figure 4A).

### Effects of SSD on Neuroinflammation and Oxidative Stress in the MePD

Nextly, we tested whether SSD could attenuated neuroinflammation and inhibiting the oxidative stress. We performed ELISA studies to measure the IL-1β and IL-6 in the MePD immediately after the behavioral tests. CMS increased the IL-1β and IL-6 levels as compared to control group, the treatment of SSD significantly decreased the IL-1β and IL-6 levels in MePD of the mice as compared to CMS group (Figures 4C,D). CMS induces significantly increased ROS production compared with the control, however, SSD treatment effectively mitigate the elevation of the ROS levels in the MePD of the mice (Figure 4E).

### Neural Plasticity in the MePD of CMS Mice After SSD Administration

**FIGURE 5:**
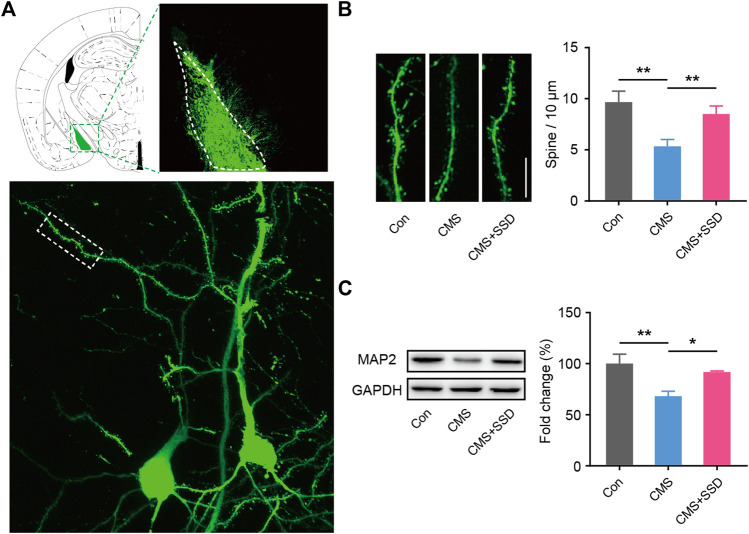
Changes in neural spine density in the MePD. **(A)** Schematic of the viral injection, and the representative neuron imaged by Z-stacked confocal microscopy. **(B)** Spine density was expressed as the number of spines per 10 μm. (*n* = 12 slices from three mice, 5–6 neuron per slice, one-way analysis of variance [ANOVA]). (Scale bar = 10 μm). **(C)** MAP2 expression was determined by a western blotting analysis. (*n* = 5, one-way ANOVA). Data are presented as the mean ± standard error of mean. *p < 0.05, **p < 0.01.

Two weeks before the end of the CMS modeling, we infected MePD neurons with AAV-CMV-GFP. We measured the spine density in these local pyramidal neuronal cells that were tagged with GFP to reveal the morphological characteristics (Figure 5A). Spines protruding from second order dendrites were separately assessed for distal dendrites. The CMS mice showed significantly decreased spine density arising from the distal second order dendrites than that in the normal control mice (Figure 5B). The spines of dendrites in the SSD-treated group showed a significant reversion of the above-stated deficit. Consistent with the abovementioned observation, the expression of the neuronal marker MAP2 was decreased in CMS mice (Figure 5C), whereas MAP2 expression was significantly increased in the MePD of CMS+SSD mice.

### Effects of SSD on Neural Firing Activity in the MePD

**FIGURE 6:**
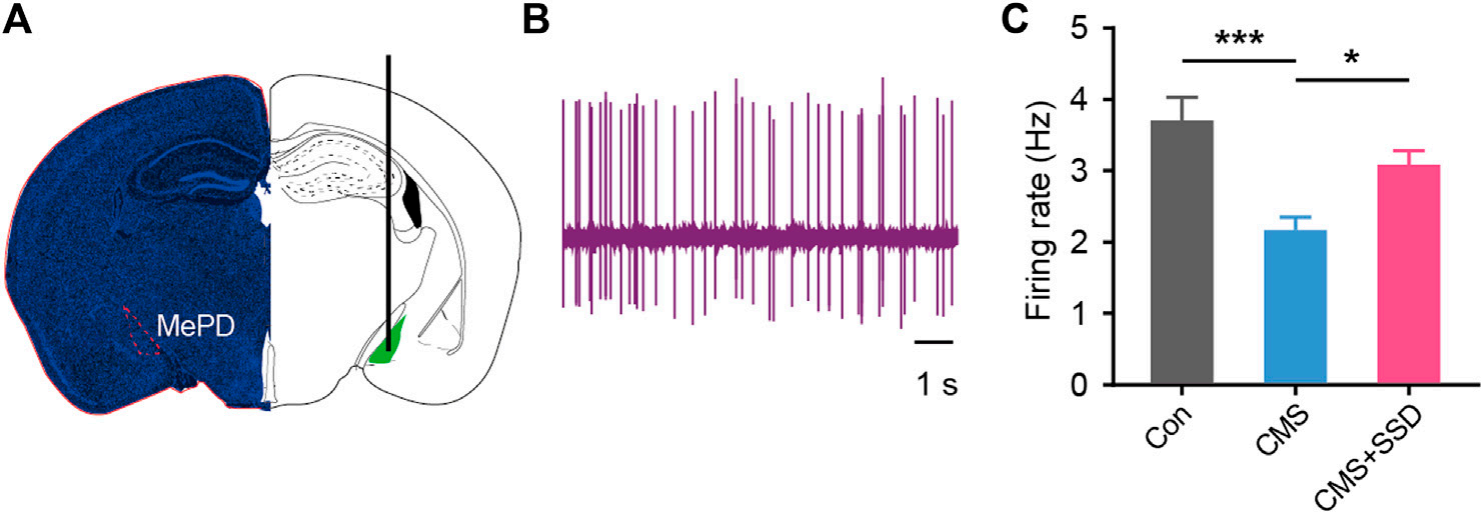
Recording of the *in vivo* activity of MePD neurons. **(A,B)** Location of the recording site and spontaneous firing activity electrophysiological properties of recorded neurons. **(C)** Statistical results of firing rate of MePD neurons from each group. (*n* = 12, one-way analysis of variance). Data are presented as the mean ± standard error of mean. *p < 0.05, ***p < 0.001.

The firing rate of MePD neurons was measured by inserting an electrode into the brain (Figures 6A,B). CMS modeling significantly decreased the firing rate of MePD neurons (Figure 6C). This decreased neuronal firing was reversed in CMS+SSD group, which indicated that the restoration of the firing properties of the MePD may underpin the effects of SSD.

## Discussion

Mood disorders, such as depression, are mainly caused by chronic stress or stressful events, with a high lifetime prevalence of reaching 20% (Patten, 2003). Sexual dysfunction, characterized by decreased sexual motivation and impaired sexual performance, is a major comorbidity in men with depression (Clayton et al., 2013) and current therapies are only partially effective, with a slow onset of efficacy, and may even lead to more severe sexual dysfunction (Atmaca, 2020). In the present study, we found that treatment with SSD can not only generate a stable antidepressant effect but also significantly alleviate sexual dysfunctions in CMS mice. We explored the central brain mechanisms underlying chronic stress-induced sexual dysfunction in mice. Pathological changes and neuroinflammation were observed in the glial and neuronal cells of the MePD of CMS mice; they may be involved in the manifestation of stress-induced sexual dysfunction. We speculated that the restoration of sexual activity by SSD may be due to its participation in the neurotrophic protection of the MePD.

Antidepressants prescribed by psychiatrists, such as serotonin reuptake inhibitors, have the well-known adverse effect of affecting sexual activity (Ishak et al., 2013; Clayton et al., 2015); these drugs may further aggravate the negative consequences on depression-induced impairment in sexual activity and affect patients’ quality of life, which could leading to patients’ resistance to medication and treatment failure. These male patients with impaired sexual function often report these sexual symptoms and seek help from andrologists, who often lack in-depth understanding of the brain mechanisms implicated in this syndrome; moreover, available treatments are mainly focused on providing symptomatic management, without a curative intent. Hence, there is an urgent need in this field for further interdisciplinary research.

The animal model of CMS has been widely used in studies on depression, which simulates various types of stress stimuli encountered by men in the current society (Brotto et al., 2001). The sexual dysfunction induced by this modeling strategy has been widely reported, such as reduced sexual motivation (Shen et al., 2020), nonorganic erectile dysfunction (Chen et al., 2019), impaired sexual behavior (Brotto et al., 2001), testicular damage (Hou et al., 2014), etc. In contrast, there is scarce information about the implications of central psychophysical mechanisms originating from the CNS. As a common clinical auxiliary therapy, TCM has been reported to have a therapeutic role in the regulation of emotions and sexual activity (Wang et al., 2019a; Ren et al., 2019). Bupleurum is the main ingredient in TCM prescriptions for the treatment of depression and sexual dysfunction (Lu et al., 2018). As the main active ingredient of Bupleurum, SSD has been reported to have antidepressant effects in rats (Li et al., 2017). However, it remains to be explored whether this effect involves modulation of sexual activity. Herein, we modeled CMS in mice and systematically analyzed the sexual motivation and performance of the CMS mice. SSD treatment improved depression-related behaviors in these mice, while also improving sexual motivation and performance. These findings are exciting and point toward the multi-modal effects of SSD.

Increasing evidence has revealed that the male reproduction system is extensively regulated by the CNS (Nadelhaft and Vera, 1996; Bancila et al., 2002), thus providing a new perspective to better understand related brain/reproductive system axis diseases. Sexual dysfunction caused by stress is not simply an symptom related to anhedonia associated with the reward system. As an instinctive behavior, sexual activity is linked to a conservative, intrinsic regulatory brain region (Kondo, 1992). Only few studies have analyzed stress-induced sexual dysfunction in terms of the pathophysiological and functional changes in the brain nuclei associated with sexual functions. As the subnucleus in the amygdala is closely related to sexual activity, MePD is evolutionarily conserved and plays an important role in arousal and the execution of human sexual behavior (Kühn and Gallinat, 2011; Seok et al., 2016). The MePD is considered to be a key integrator of systemic arousal and sexual sensory stimulation and is involved in both emotional regulation and sexual performance (Rasia-Filho et al., 2012; Bergan et al., 2014; Holder and Mong, 2017), thus we explore its potential contribution in the psychological stresses-related sexual dysfunction.

Astrocytes are the most abundant cells in the CNS (Namihira and Nakashima, 2013) and have been found to play an important role in stress-induced behavioral abnormalities (Jun et al., 2018); however, their function in the MePD remains largely unknown. During CMS modeling, astrocytes, which are involved in the initiation of neuroinflammation, are activated in the hippocampus (Du Preez et al., 2021). However, regarding the astrocytes in the MePD, the depressive-like symptoms are associated with a decreased density and hypofunction of astrocytes, which is expected to contribute to synaptic dysfunction in the MePD. Our results showed that the number, volume, and protrusion length of MePD astrocytes were decreased in male mice experiencing chronic stress, thus reducing the plasticity of astrocytes in local brain regions. SSD had a protective effect on astrocytes, which could antagonize the CMS-induced damage to astrocytes and enhance their plasticity. Stress can cause a vicious cycle, increasing microglial activation and neuroinflammatory dysfunction (Calcia et al., 2016). In this study, under condition of CMS, microglial activation and neuroinflammation were significantly increased in the MePD. CMS promoted proinflammatory microglial activation, microgliosis, and increased the concentration of inflammatory markers, such as interleukins, and increased ROS expression in the MePD. The effects of SSD in inhibiting microglia activation and neuroinflammation are consistent with those reported in previous reports (Su et al., 2020).

Chronic stress can significantly affect the synaptic plasticity and neuronal activity of the amygdala subnucleus (Marcuzzo et al., 2007), which suggests that CMS may produce changes in synaptic plasticity-related neural functions in the MePD. After observing the morphology of local neurons in the MePD, we found that the density of dendritic spines in the pyramidal neurons decreased significantly, suggesting weakened connections between the neurons. The electrophysiological results further showed impaired neuronal activity in the MePD, revealing that chronic stress caused synaptic plasticity-related impairment in neural functions in the MePD, and that SSD treatment can effectively reverse these changes. During the above processes, astrocytes are involved in the regulation of local neural activity (Chih and Roberts, 2003). Changes in astrocyte polarization were implicated in the abnormal neural information processing. Deepening understanding of the function of astrocytes and SSD’s psychopharmacological target in the MePD are worthy of future research focus. In addition, the MePD receives projections from upstream brain regions and sends signals down to its related brain regions, the MePD-related circuit mechanisms implicated in this brain-related sexual dysfunction still warrant further investigation.

Taken together, our results have shown for the first time that SSD has a dual therapeutic effect on stress-induced depression and stress-induced sexual dysfunction. Our data provide significant evidence in support of the presence of glial and neural pathologies in an animal model of brain-related sexual dysfunction diseases (BRSDD). These findings broaden our psychopharmacological insights into the role of SSD and lay the foundation for the development of novel potential therapeutic strategies to treat BRSDD.

## Data Availability

The raw data supporting the conclusions of this article will be made available by the authors, without undue reservation.
